# Subjective Risk Assessment and Perception in the Greek and English Bakery Industries

**DOI:** 10.1155/2009/891754

**Published:** 2009-10-27

**Authors:** Evangelos C. Alexopoulos, Zafira Kavadi, Giorgos Bakoyannis, Sotiris Papantonopoulos

**Affiliations:** ^1^Department of Public Health, Medical School, University of Patras, 26500 Patras, Greece; ^2^School of Environment & Life Sciences, University of Salford, Manchester M5 4WT, UK; ^3^Department of Hygiene and Epidemiology, Medical School, University of Athens, 11527 Athens, Greece; ^4^Department of Production and Management Engineering, Faculty of Engineering, Demokritos University of Thrace, 67100 Xanthi, Greece

## Abstract

Several factors influencing risk perception in the area of occupational health and safety are known, but there is still lack of a full understanding of the ways in which people characterize risk. This study aimed to provide an insight of employee risk assessment and perception in the bakery industry. 87 British and 64 Greek employees in two comparable bakery companies were asked to estimate and evaluate hazards at their workplace. The participants' judgments of 12 hazards—according to 7 risk aspects—were collected and analyzed. Subjective assessment on important occupational hazards included handling heavy loads, repetitiveness, high temperatures, high rate of work, stressful deadlines, and noise. Although limited in the population involved, our findings revealed strong cross-national differences in employee risk perception of specific groups of hazards in the bakery industry. Additional interviews revealed evidence that Greek employees' risk perception depends mostly on work experience while British employees were aware of risks due to company health and safety policy, recognizing that safety is the responsibility of both the management and the worker. Cross-national (cultural) factors that influence workforce risk perception and attitudes towards safety have to be taken into account by technical experts and policy makers in the designing of prevention strategies and risk communication.

## 1. Introduction

Risk is intuitively familiar and broadly applicable in everyday life. Exposure to risk varies between societies, depending among other factors, on culture, education, economic conditions, social and environmental injustice, technological infrastructure, public health priorities, and natural hazards.

Risk assessment evaluates hazards by measuring/assessing the probability and the severity of the associated adverse effects. Research on risk perception has shown that the probability and severity of adverse effects are not the only components that most people use as yardsticks for perceiving and evaluating risks, but also the context in which those risks are experienced. The dependence of risk perception on the circumstances in which those risks are experienced is not random but rather follows certain principles that have given rise to systematic psychological investigation. The importance of risk perception research lies in the prediction, risk communication, and optimization of interventions on risk reduction [1].

Furthermore, most studies investigating the way people perceive and respond to risks have emphasized cognitive factors, although emotional, economic, social, and cultural factors have also been advocated in this field. Important contributions to our current understanding of risk perception have come from sociological and anthropological studies [2, 3]. Investigators have demonstrated cross-cultural differences in the perception of risky activities that pose threats to health and safety [4–6]. Assessing workforce perceptions of risk is of importance in order to develop a proper safety culture. Risk perception research has been criticized for insufficient analysis of the causal relationships between risk factors and perceived risk. Studies on risk perception have shown that personality characteristics have to be examined at individual and cultural level [7, 8].

People perceive risk in different manners in various situations and such perception is influenced by early experiences, education, personal beliefs, attitude of coworkers, and culture [9–11]. Researchers emphasized the importance of various social and institutional factors to risk perception, factors which are ultimately combined by human judgment [12, 13]. Experience holds a vital role in risk perception since, for example, misleading experiences may underlie an individual's tendency to believe that he or she is personally immune to many hazards [14–17].

In sum, research has provided a lengthy list of supporting circumstances or qualitative factors that have some bearing on risk perception and factor analysis reduced these lists to a few important compound factors. Such studies have been conducted in Austria, Germany, Great Britain, the Netherlands, and the USA [18]. Renn and Rohrmann [18] have identified the following factors as particularly relevant: the familiarity with the risk source, the voluntary acceptance of the risk; the ability to personally control the degree of risk, whether the risk source is capable of causing a disaster (catastrophic potential), the certainty of fatal impact should the risk occur (dread), the undesired impact on future generations, the sensory perception of danger, the impression of fair distribution of benefit and risk, the impression of reversibility of the risk impact, the congruence between benefactors and risk bearers, the trust in state-operated risk control and risk management, the experience (collective and individual) with technology and nature, the reliability of information sources, and the clarity of information on risk.

Rohrmann and Chen (1999) [19] have conducted a cross-cultural study of risk perception in China and Australia in order to analyze the cognitive structure of judgments about the magnitude and acceptability of risks to which individuals and specified societal groups are exposed. The results showed that there was considerable cross-national variation in risk perception, and groups affiliated with particular professional orientations differed in their judgment and evaluation of hazards as well. Furthermore, the research hypothesis that individual risk acceptance is higher in China was not confirmed. A major disparity between the two country data was that the Chinese respondents seemed to be less prepared than the Australian ones to accept risks in principle (there was no difference in the mean of risk magnitude ratings).

The objectives of this study were

- to investigate subjective risk assessment of British and Greek employees in the bakery industry,
- to examine and analyze the structure of judgments employees made when they are asked to evaluate specific hazardous activities in the bakery industry,
- to compare the judgments in terms of individual, employment, organizational, and cross-national factors.

## 2. Subjects and Methods

### 2.1. Study Design and Data Collection

We have chosen bakery industry based on accessibility in both British and Greek companies, and we have succeeded to find two very similar—in terms of the number of employees, infrastructure, and scale of production—bakery companies. In both companies a member of the research team (ZK) was employed as a trainee occupational health and safety assistant advisor for a three-month period. During this training course the data collection took place. All employees and managers knew that ZK was collecting data for a study. We examined differences in working conditions through direct observation and interviews with the occupational health and safety managers, based, where possible, on measurements of risk factors. Data from the study participants were collected through note taking, in a rather free-association form. In both countries, employees were observed during different hours of the working day.

Additional data were collected in a three-month period by the use of an anonymous self-administrated questionnaire and a semistructured interview. The questionnaire consisted of three sections including information on personal and employment characteristics, questions on exposure assessment to specific job hazards, and question on risk definition and questions on rating twelve occupational hazards according to seven qualitative risk aspects. The hazards were chosen to be relevant to bakery industry based on both literature review and previous work by researchers on risk assessments in bakeries. The choice of risk aspects was based on literature review [12, 13, 18, 19]. The questionnaire was translated in Greek language and checked via back translation by two bilingual scientists. The variables, scales, and instructions were identical to the English version, and any severe impact of the translation in results was unexpected. Prior to the study, the questionnaire was tested for comprehensibility and relevance on five employees in each country. One limitation of the self-administered questionnaires is that some questions may be misunderstood or be untruthfully answered. Another drawback is that there might be higher percent of missing answers.

Individual characteristics and work history included questions on age, gender, level of education, duration of employment, and job title held. Questions on exposure assessment involved exposure to noise, chemicals, high temperature, lifting and carrying heavy loads, awkward working postures in which the back is bent or twisted, and repetitive movements. Psychological exposure distinguished control over work (rate of work and deadlines) and task variety (monotonous work). For both the physical and psychological factors a five-point scale was used ranging from never to always.

In the last section, employees were asked to define what risk is. For this, three possible answers were available: the frequency of a hazard, the severity of its consequences, and that risk definition depends on the hazard.

The last section of the questionnaire also examined how employees judge a number of different hazards with respect to seven qualitative risk aspects (i.e., frequency, controllability, knowledge, dread, voluntariness, familiarity, and catastrophic potential). Employees were asked to rank the following twelve hazards: (1) contact with electricity, (2) hit by falling objects, (3) exposure to fire, (4) slip/fall on the level, (5) falling from height, (6) cut/bruising, (7) contact with moving vehicles, (8) contact with machinery, (9) exposure to noise, (10) exposure to harmful substances, (11) lifting/moving heavy load, and (12) strenuous working postures. Respondents were requested to rank these hazards according to how often they encounter these twelve hazards as they are at work (frequency) and based on the level of their knowledge and their feeling of control over these hazards. A ranking of 1 (according to a certain risk aspect) was considered as the major hazard and a ranking of 12 as the minor. Participants also ranked these hazards according to their feeling of familiarity, dread (worrying about), voluntariness (taking precautions), and the catastrophic potential (harming many people).

In addition, three interviews in each country were carried out as a supplementary way to investigate the research questions [20–24]. An interview schedule was drawn up consisting of topics with some specific open questions under each topic heading. This schedule was then used as a guide for the researcher rather than a definite and exhaustive list of questions [20–24]. The interview format chosen was the semistructured interview. This format was found to be the most appropriate for this study [20–24].

### 2.2. Statistical Analysis

Self reported occupational risk factors were compared between Greek and English employees. These comparisons were performed using the Mann-Whitney test, and the Bonferroni adjustment was used to account for the multiple tests. For the analysis of risk definition, multinomial logistic regression was used [25, 26]. Risk definition had three levels: frequency, severity of its consequences, and dependence on the hazard. A backwards elimination procedure based on likelihood ratio test was applied for variable selection (removal criterion 0.05 level of significance). Age and job were included in each step of the procedure and in the final model regardless of their level of significance.

For each of the 12 occupational hazards, a score was generated by summation of ranks over the qualitative characteristics controllability, knowledge, dread, familiarity, and catastrophic potential, as an index of risk perception. These scores were included in a factor analysis in order to reduce the dimensionality and to identify underlying latent, immeasurable variables, the factors [27]. The principal component method was used for estimation and the Varimax rotation was applied to help with the interpretation of the factors. A loading value larger than 0.5 was considered significant. The number of the factors was determined by the Kayser criterion, as the number of eigenvalues was larger than their average value of 1 (since the correlation matrix was used). Factor scores generation was based on regression method. The resulting factors were included in a multivariate analysis of variance as dependent variables. We used MANOVA since there was no evidence of heterogeneity of covariance matrices from Box's test of equality (*P* = .684). Moreover, we conducted a graphical check of multivariate normality which was based on the standardized distance (calculated in R package) of the vector of factors for each subject from the mean vector, that has shown that factors followed a multivariate normal distribution. For variable selection, a backwards elimination procedure was applied based on Wilks' test (removal criterion 0.05 level of significance). Risk definition and job title were included in each step of the procedure and in the final model regardless of their level of significance. SPSS for Windows was used for all statistical calculations.

### 2.3. Interviews

Data from interviews were analyzed following the method of thematic analysis. The data were examined repeatedly to look for broad themes. Items were extracted and collected into the emergent themes. Thus, large amounts of data were reduced into smaller units. The analysis began by building an array of categories from systematic inspection of the data. Categories were generated to fit or provide an interpretable description of the data. The process of categorization was continued by checking whether the remaining part of the interviews fitted into the already formed categories or suggested new categories. The procedure was terminated when the coding of the data no longer contributed any further insights. The strength of this way of analysis lies in its emergence. It has the great advantage that it is not bound by predetermined categories but it is free to search for categories and concepts that appear meaningful to participants; it allows participant’ views to emerge [20–24].

Standardising the interview process over six interviews was problematic due to the nature of the method. The only standardization attained was to ensure that opening remarks and explanations were the same for all participants and subsequently cover all topic areas. The fact that the interviews were participant-led often meant that it was not possible to go through the topics in the same order for each participant, as they were presented in a natural way during the interview.

The sample was random. Six individuals, three for each country, were asked to give an interview during this study. One among the three individuals was a supervisor whereas the other two were workers. This number was found to be adequate for this study since, as already mentioned, the interview method was primary used as a complementary technique to collect information that supported the data obtained by questionnaires. In the UK, two participants were male and one woman. Ages ranged from 25 to 45 years old. Two of the participants had been in the industry for 5 and 11 years while the third one for 1 year. In Greece, two participants were women and one man. Ages ranged from 30 to 48 years old. Two of the participants had been in the industry for 14 and 19 years and the third one for 5 years.

## 3. Results

### 3.1. Description of the Companies

#### 3.1.1. The British Bakery

There were 350 employees in the British bakery. The working areas could be divided into two levels of risk. The high-risk working area included departments where activities and process raised more workplace risks in contrast to other departments. Company's safety management included a number of safety features, implemented by the health and safety officers. Written risk assessment, manual handling assessment, information leaflets about injuries and treatments, first-aid brochures, and accidents monitoring were available. In addition, the British bakery provided training for employees on health and safety rules. As a result, most British employees used their appropriate personal protective equipment (PPE) and washed their hands before going in the high-risk working area. Health and safety notes existed in all departments such as instructions for manual lifting or using PPEs and yellow warning signs for slippery floors. There was also a fire training course once per week as well as a more extensive fire training course every month. The fire training course is of particular importance for this industry due to the presence of high flammable materials (polyester). In general, in terms of health and safety, the entire industry atmosphere was supportive towards a safety attitude. Despite this supportive atmosphere, some employees did not make use of the PPEs.

#### 3.1.2. The Greek Bakery

There were 275 employees in the Greek bakery. The departments of the company included engineering, production packing, and the bakery store. The health and safety management of the industry included a number of features. The safety management of the company included written risk assessment and accidents monitoring. The industry offered educational courses every semester mainly towards food safety and less on occupational health and safety. There was also fire training once per week. In addition, there was periodic examination of the employees' health from an external medical doctor as well as inspection of the company facilities and workplace from inspectors of the Ministry of Labor. A relatively small number of safety notes were observed in the different departments; however, the lack of warning notes was obvious in some places of high risk. There was more attention in food safety rather than in occupational health and safety. The company had also taken some measures to minimize risks. For example, it was reported that sound absorbing wall materials were installed to reduce noise levels. In comparison to the British company, more employees avoided making use of the appropriate PPE.

### 3.2. Response and Baseline Characteristics

One out of three employees randomly selected were asked to participate in the study. The response rate was 79% (151/190 respondents). Nonrespondents did not return questionnaires (6%) or they did not complete all the questions (15%) and were excluded from the analysis. The respond rate was higher in Greece (91%) compared to the UK (72%). The sample was selected at random from different departments, consisted predominantly of men (67.5%) with ages ranging from below 25 to over 55 years. Most respondents had a low educational status (i.e., up to nine year's total education). Job titles represented were workers (77.5%), supervisorsorteam leaders (9.9%), and managers (12.6%). In both countries, managers oversee supervisors and work mainly outside the production line. Table 1 shows the basic characteristics between British and Greek employees. There were differences between the two samples with respect to gender, educational level, and duration of employment.

### 3.3. Physical and Psychological Load

The most important occupational hazards in our study involved heavy loads, repetitiveness, high temperatures, high rate of work, stressful deadlines, and noise. Employees in the Greek company reported significantly higher exposure to noise, harmful substances, and rate at work while British reported more stressful deadlines (Table 2). Females reported higher exposure to repetitive movements and higher rate of work than males. As expected, workers reported higher exposure than supervisors with respect to noise, temperature, heavy loads, painful postures, and repetitive movements. Finally, employees with less than 10 years of employment reported more repetitive movements than those with over 10 years in the companies.

### 3.4. Risk Definition

Employees were asked to define risk. In Table 3, results from the multinomial logistic regression analysis of risk definition are shown. The results showed that supervisors/managers, English employees, and employees with higher exposure to risks were better aware of risk definition, defining the risk by the frequency of a hazard or the severity of its consequences compared (resp.) to workers, Greek employees, and employees not highly exposed to risk who answered that the risk depends on the hazard.

### 3.5. Risk Perception

Employees were asked to rate twelve hazards with respect to risk aspects. All twelve scores that were generated by summation of ranks over the qualitative characteristics controllability, knowledge, dread, familiar, and catastrophic for each of the 12 occupational hazards, as indexes of risk perception included in a factor analysis (Table 4). We end up to five factors named as General hazards, Ergonomics-related hazards, Hygiene-related hazards, Equipment-related hazards, and Transportation-related hazards. Multivariate tests for the effect of independent variables on the factors have shown that job title (*P* = .148) and Risk definition (*P* = .243) did not exhibit significance while nationality (*P* < .001) remained as the only important variable after the multivariate analysis of variance. More specifically, in three out of the five resulting factors there was evidence of real effect of nationality in bakery employees (Table 5). Age and sex did not account for differences in cultures. After adjusting for both variables there was significant effect of nationality.

### 3.6. Interviews' Results

Due to the limited number of subjects this qualitative approach had been only focused on the employees' perception and judgments about the risk with regard to their work activities in the industry.

#### 3.6.1. The British Bakery (3 Employees)

Two employees perceived the risks as controllable by being aware of and following health and safety directives; two also perceived themselves as responsible to implement safety measures in order to prevent accidents in their work environment; one of the three employees understands the risks and promotes ways for the development of health and safety performance measures; all three employees feel that they are aware of the risks and that they are protected from accidents because of the management actions of the industry towards safety (e.g., safety warning notes, injury prevention leaflets).

#### 3.6.2. The Greek Bakery (3 Employees)

Two employees perceived the risks as controllable because of their work experience; All three employees indicated that the responsibility for risk control measures lies to themselves by being cautious; one understood the risks and considered that it is his or her responsibility to inform the new employees about them; one perceived the risks as controllable because of the health and safety rules applied by the industry.

## 4. Discussion

This study provides evidence that the most important occupational hazards in the bakery industry involve handling heavy loads, repetitiveness, high temperatures, high rate of work, stressful deadlines, and noise. It also showed that supervisors/managers and English employees were better aware of risk definition. Of most importance was that our study examined the judgments employees made when they were asked to evaluate specific hazardous activities in the bakery industry. Our results revealed strong cross-cultural differences in employee risk perception of specific groups of hazards.

Two similar bakery companies were selected in Greece and England. Some differences were nonetheless anticipated in working conditions in the Greek and British bakeries in terms of occupational hazards. To estimate the true differences of the working conditions in the bakery environments, we analyzed data from the subjective risk assessment of the employees, information of health and safety managers, and personal observations. We concluded that the companies were highly similar in terms of occupational health and safety even though Greek employees reported more physical exposure and underwent training and education towards occupational safety at a lesser extent.

The representativeness of the sample for studying cultural differences is questionable in some extent because besides white and black British, the British bakery included a few employees from other national cultures like Indians. The question applies, however, to many other cross-cultural studies. In their study of risk perception in China and Australia, Rohrmann and Chen (1999) [19] conceded that representative samples of these groups and countries were neither possible nor intended as both countries are very complex multicultural societies (which to reflect was far beyond their research) and that their project did not claim to compare Australians with Chinese but, rather, to use societal distinctions in order to elucidate typical cross-cultural differences in risk evaluation. A similar assertion is made by the current study.

Another issue is the adequacy of the sample for studying cultural differences. We are aware that sample sizes are relatively small in both countries and furthermore comparing just two companies is a rather weak basis for comparing cultures. But since we have comprehensively tested the possible differences, we are confident that in a great extent the differences found might at least partly be due to a psychological tendency to rate identical hazards in a different way in the two companies. This tendency might reflect individual-level cultural differences or in psychological functioning of the participants. Our findings are supported by other studies, which have underlined the cultural variations in risk perception [10, 28–30].

The education/training might be responsible for some differences. Education/training affects attitudes towards hazards and influences the perceived hazard. The effectiveness of warning sites also assumed to have played a role in educational process [31–33]. Exposure to fire was reported as the least frequent and unfamiliar hazard by the employees in both companies. While British employees perceived it as more catastrophic, Greek employees perceived this risk as the most dreadful. Frosdick [34] has noted that people tend to fear the risk involved in an activity that they are not familiar with. It seems that training holds an important role in some risk aspects and may be responsible for modifying risk perception related to dread and familiarity factors in both countries. If the activity involved is something that the person is familiar with, the individual usually has substantial amount of personal experience and knowledge to judge the situation [3, 6]. A study shows that differences in experience of risk activities can explain individual variability in risk assessments but their relationship depended on whether the risk experiences were voluntary or not [35].

Another worth mentioning finding was that while Greeks perceived common hazards as more catastrophic, they were less worried about them, take fewer precautions, and consider them as being outside their control compared to British employees. Surveys have shown that people worry less about those risks that they feel they are able to control [11, 36].

Nisbett and Masuda [37] showed that Americans and northern Europeans think that they can control events because they know the rules that govern the behavior of objects. On the other end of a cultural spectrum, East Asian cultural groups tend to believe that events are highly complex, are determined by many factors and, inevitably, are less controllable. Eastern and southern Europeans (including the Greeks) tend to lie within these extremes. Furthermore, the Greek working class shall be expected to embrace stronger fatalistic beliefs than Europeans since they consider, in a greater extent, some occupational hazards as outside their control. Therefore, the observation that Greek employees consider some risky situations outside their control goes along with such fatalistic beliefs.

Interviews confirmed that Greek employees' risk perception depends on work experience rather than education, training, and other management actions aiming on risk control and prevention. Greeks emphasize that risk management relies on personal responsibility and is associated with work experience. Perhaps lack of trust may influence this statement. Greek employees are often suspicious against management actions. Trust has argued as a factor that influences perception, especially for hazards about which people did not possess much knowledge, but other researches has shown that it is not shown to be powerful [38, 39]. British employees are aware of risks due to health and safety policy recognizing that safety is the responsibility of both management and the worker together. This finding showed that company policy affects employees in different extents and is consistent with other studies that found that the health and safety management structure appears to influence the way individuals perceive the risks and the options they have for their control [40].

Risk perception is hard to understand. Several factors are known to influence it but there is still lack of a full understanding of the ways in which people characterize risk. This study tried to provide an insight of employees' views and their risk assessment in the English and Greek bakery companies. Although limited in the population involved, our findings provide clues of cultural variations in risk perception of occupational hazards. Combined with education/training, these differences are responsible for variations found in risk perception. In-depth psychological research towards a model based prediction of the behavior in occupational health settings is needed in order to explain individual, group, or cultural influences [41]. Motivation, beliefs, and values are important elements of interventions towards health and safety. Effective interventions in both organizational and policy levels have to take into account motivation, satisfaction, and performance of employees based on knowledge of individual and situational characteristics [42–45].

## 5. Conclusions

This study provides evidence of subjective estimates of the most important occupational hazards in the bakery industry. These include handling heavy loads, repetitiveness, high temperatures, high rate of work, stressful deadlines, and noise. It also showed that supervisors/managers and English employees were better aware of risk definition. Of most importance was that our study examined the judgments employees made when they were asked to evaluate specific hazardous activities in the bakery industry. Our results revealed strong cross-national differences in employee risk perception of specific groups of hazards. Technical experts and policy makers have to take into account these cultural variations in decision-making, the design of prevention strategies, and risk communication. In addition, knowledge of workforce risk perception and attitudes towards safety is necessary for the development of a safety culture, where each person accepts responsibility for safe work. It is obvious that further research is needed to investigate in what level the cultural variation in risk perception affects the implementation of laws like European Directives on occupational health and safety, and more importantly, if these directives could influence and modify these variations.

## Figures and Tables

**Table 1:** Personal characteristics of British (*n* = 87) and Greek (*n* = 64) employees in bakery industry.

	British		Greeks	
	*n*	%	*n*	%
Age				
>35 years	49	56.3	27	42.3
≤35	38	43.7	37	57.8
Gender*				
Male	76	87.4	26	40.6
Female	11	12.6	38	59.4
Educational level*				
Lower	64	73.6	56	87.5
Higher	23	26.4	8	12.5
Occupation				
Managers	13	14.9	6	9.4
Supervisors	12	13.8	3	4.7
Workers	62	71.3	55	85.9
Duration of employment*				
>10 years	19	21.8	24	37.5
≤10	68	78.2	40	62.5

*X^2^, *P* < .05.

**Table 2:** Subjective risk assessment by Greek and English employees.

Exposure	Median score**		
	English	Greek	*P*-value
Physical/chemical/ergonomic factors			
Noise	2	3	<0.001*
High temperature	2	3	0.765
Chemicals	0	2	<0.001*
Lifting and carrying heavy loads	3	3	0.694
Awkward working postures	2	2	0.012
Repetitive movements	3	3	0.017

Psychosocial/organization			
Rate of work	2	3	<0.001*
Deadlines	3	2	<0.001*
Monotonous work	2	2	0.477

*Significant at 0.0056 level of significance (Bonfferoni correction).

**Ranging from 0 (=never) to 4 (=always).

**Table 3:** Multinomial Logistic Regression analysis for risk definition*.

	OR_frequency_**	95% C.I.	OR_severity_**	95% C.I.
Age	0.75	0.50–1.12	1.12	0.65–1.95
Job title				
Workers	1.00	—	1.00	—
Supervisors or managers	**3.84**	**1.14**–**12.95**	1.51	0.30–7.66
Nationality				
Greek	1.00	—	1.00	—
English	**4.65**	**1.98**–**10.93**	2.16	0.69–6.78
Physical exposure score	**1.12**	**1.01**–**1.24**	1.01	0.88–1.16

*Frequency of hazard” or “severity of its consequences” versus “it depends on the hazard.”

**OR_j_: Odds Ratio of defining risk as j versus “it depends on the hazard,” adjusted for the other covariates.

**Table 4:** Factor loadings after Varimax rotation.

Hazard score*	Factor 1	Factor 2	Factor 3	Factor 4	Factor 5
Contact with electricity	−**0.69**	0.21	−0.18	−0.16	−0.29
Hit by falling objects	−0.09	**0.53**	0.18	−**0.59**	−0.05
Exposure to fire	−**0.70**	0.14	−0.19	−0.16	0.07
Slip/fall on the level	0.27	0.25	**0.74**	−0.05	−0.11
Falling from height	−0.46	0.47	0.05	−0.03	0.29
Cut/bruising	0.20	−0.23	**0.61**	0.20	−0.42
Contact with moving vehicles	−0.02	0.02	−0.05	0.09	**0.92**
Contact with machinery	0.20	0.02	−0.03	**0.81**	0.04
Exposure to noise	**0.77**	0.14	−0.14	0.14	−0.16
Exposure to harmful substances	0.28	−0.09	−**0.75**	0.20	−0.19
Lifting/moving heavy load	0.02	−**0.89**	−0.06	0.09	0.00
Strenuous working postures	**0.55**	−**0.53**	−0.11	−0.33	−0.02

*Sum of the rank (ties were not allowed) of each hazard over qualitative risk characteristics controllability, knowledge, dread, familiar, and catastrophic potential.

**Table 5:** F-tests for the effect of nationality on each factor.

	F	*P*-value
Factor 1: General hazards	17.28	<**.0001**
Factor 2: Ergonomics	1.02	.315
Factor 3: Hygiene	4.04	**.046**
Factor 4: Equipment	0.30	.583
Factor 5: Transportation	5.33	**.022**
